# Supplementary material for: Use of AbobotulinumtoxinA in Adults with Cervical Dystonia: A Systematic Literature Review
Source: Toxins (Basel). 2020 Jul 24;12(8):470. doi: 10.3390/toxins12080470 (PMC7472382; doi:10.3390/toxins12080470)
Supplement: Supplementary file 1 [file toxins-12-00470-s001.pdf]

Supplementary Material: Use of AbobotulinumtoxinA in Adults with Cervical Dystonia: A Systematic Literature Review

Table S1. Database search strategy.

Database	Search String
**PubMed**	(“abobotulinumtoxinA” [Supplementary Concept] OR “abobotulinumtoxinA” [All] OR “Dysport” [All] OR “abobotulinum toxin-A” [All] OR “abobotulinum toxin A” [All] OR “abobotulinum toxinA” [All] OR “aboBoNT-A” [All] OR “aboBoNT A” [All] OR “aboBoNTA” [All] OR “A/Abo” [All] OR “AAbo” [All] OR “A Abo” [All] OR “BoNT-ABO” [All] OR “BoNT ABO” [All] OR “BoNTABO” [All]) AND (“Cervical dystonia” [All] OR “spasmodic torticollis” [All] OR “torticollis” [All] OR “laterocollis” [All] OR “anterocollis” [All] OR “retrocollis” [All]) AND (“Adult” [Mesh])
**Cochrane**	TX (“abobotulinumtoxinA” OR “Dysport” OR “abobotulinum toxin-A” OR “abobotulinum toxin A” OR “abobotulinum toxinA” OR “aboBoNT-A” OR “aboBoNT A” OR “aboBoNTA” OR “A/Abo” OR “AAbo” OR “A Abo” OR “BoNT-ABO” OR “BoNT ABO” OR “BoNTABO”) AND TX (“Cervical dystonia” OR “spasmodic torticollis” OR “torticollis” [All] OR “laterocollis” OR “anterocollis” OR “retrocollis”) and SU adult
**Embase**	(“abobotulinumtoxina” OR “dysport” OR “abobotulinum toxin-a” OR “abobotulinum toxin a” OR “abobotulinum toxina” OR “abobont-a” OR “abobont a” OR “abobonta” OR “a/abo” OR “aabo” OR “a abo” OR “bont-abo” OR “bont abo” OR “bontabo”) AND (“cervical dystonia” OR “spasmodic torticollis” OR “torticollis” OR “laterocollis” OR “anterocollis” OR “retrocollis”) AND ([young adult]/lim OR [adult]/lim OR [middle aged]/lim OR [aged]/lim OR [very elderly]/lim)

Table S2. GRADE approach on interpreting methodological quality.

Underlying methodology	Quality
Randomized trials; or double-upgraded[a] observational studies	High
Downgraded[b] randomized trials; or upgraded[a] observational studies	Moderate
Double-downgraded[b] randomized trials; or observational studies	Low
Triple-downgraded[b] randomized trials; or downgraded[b] observational studies; or case series/case reports	Very low

[a]Factors that may increase the quality level: large magnitude of effect; all plausible confounding would reduce a demonstrated effect or suggest a spurious effect when results show no effect; dose-response gradient, [b]Factors that may decrease the quality level: limitations in design and implementation suggesting high likelihood of bias; indirectness of evidence; unexplained heterogeneity or inconsistency of results; imprecision of results (wide confidence intervals); high probability of publication bias.
